# An empirical study on exercise addiction and grit among college students: based on 35 exercise sessions

**DOI:** 10.3389/fpsyg.2026.1755715

**Published:** 2026-05-20

**Authors:** Jiahao Jiang, Yating Huang, Yuanbin Sang, Xinyu Zhou, Shaofei Hou, Lun Li

**Affiliations:** School of Physical Education, China University of Geosciences (Wuhan), Wuhan, China

**Keywords:** 35 running sessions, college students, empirical study, exercise addiction, grit

## Abstract

**Purpose:**

This prospective observational study used the number of mandatory running sessions as a core between-subject variable (three groups: < 35 sessions, = 35 sessions, > 35 sessions) and measurement time as a within-subject variable (pre-test, post-test). It mainly investigated two issues: first, between-group differences in exercise addiction tendency and grit among college students with different frequencies of mandatory running; second, the correlation characteristics between exercise addiction tendency and grit, so as to provide empirical evidence for college physical education practice.

**Methods:**

A total of 1,611 college students (males: *n =* 930, females: *n =* 681, aged 18–22 years) were enrolled and divided into three groups by the number of mandatory running sessions: < 35 sessions (*n =* 380), = 35 sessions (*n =* 1,132), and > 35 sessions (*n =* 99). All participants joined regular campus mandatory running activities, with 35 sessions set as the compliance threshold, and no extra experimental interventions were applied. The Shapiro–Wilk test was used to examine data normality, and the Levene test to verify homogeneity of variance. Standard ANOVA was used for homogeneous data, and Welch ANOVA for heterogeneous data. Mixed-design ANOVA was conducted to test the main effects of group, main effects of time, and time × group interaction, accompanied by appropriate post-hoc tests. Correlation correction, sensitivity analysis, and Cronbach’s *α* coefficient were adopted to ensure methodological rigor.

**Results:**

Baseline scores of exercise addiction tendency and grit in the > 35 sessions group were significantly higher than those in the other two groups, with no significant differences between the latter two. No significant pre-to-post changes were found in the Exercise Addiction Inventory (EAI) in any group. For the Original Grit Scale (Grit-O), marginally significant pre-to-post differences were detected in the > 35 sessions and < 35 sessions groups, while no significant difference was observed in the = 35 sessions group. The time × group interaction was not significant for EAI but significant for Grit-O. There was no substantial intrinsic correlation between exercise addiction tendency and grit; their weak correlation resulted from between-group differences. The number of running sessions showed a weak linear positive correlation with both indicators. Both scales presented good internal consistency (*α* > 0.7).

**Conclusion:**

There are significant between-group differences in exercise addiction tendency and grit among college students with different numbers of mandatory running sessions. The baseline psychological traits of the high-frequency running group (> 35 sessions) are significantly higher than those of the medium- and low-frequency groups. Only the high-frequency group shows an improving trend in grit, and the number of running sessions has a significant moderating effect on grit enhancement, whereas the change pattern of addiction tendency is not affected by running frequency. Exercise addiction tendency and grit have no substantial intrinsic connection; their apparent correlation arises from superimposed between-group differences. The number of running sessions shows a linear dose–response relationship with both indicators. This study provides empirical references for optimizing campus running intervention programs and cultivating psychological qualities among college students.

## 1 Introduction

In contemporary society, rising health awareness and lifestyle changes have made exercise a beneficial approach for college students to promote physical and mental health (Herbert et al., 2020), improve academic performance (Buckworth and Nigg, 2004), and enhance quality of life (Zhou and Zhou, 2022), attracting extensive social attention. However, physical inactivity is still prevalent among college students (Costa et al., 2017; de Sousa, 2011; Ghimire and Lamichhane, 2024). Guiding students to form positive and sustainable exercise habits is necessary for individual all-round development and crucial for building a healthy campus and improving education quality (Keating et al., 2005). The formation and maintenance of exercise behavior require persistent motivation, perseverance, and identification with exercise goals (Teixeira et al., 2012). As core psychological variables, grit and exercise addiction tendency have become the focus of researchers.

Duckworth et al. (2007) first systematically studied grit and defined it as perseverance and passion for long-term goals. Despite academic controversies such as conceptual overlap with conscientiousness (Credé et al., 2017), cross-cultural applicability of measurements (Dijksma et al., 2022), and context-specific theoretical foundations (Lee et al., 2023), grit is of great significance for individual development, especially for college students (Duckworth, 2016). Grit is significantly associated with educational attainment (Duckworth et al., 2007), age (Eskreis-Winkler et al., 2014), growth mindset (Tucker-Drob et al., 2016; Yeager et al., 2016), optimistic explanatory style (Robertson-Kraft and Duckworth, 2014), self-control (Duckworth and Gross, 2014), delay of gratification (Meriac et al., 2015), goal commitment (Hill et al., 2016), self-efficacy (Mark et al., 2015), teacher-student and parent–child relationships (Datu, 2017), number of running sessions (Crosby et al., 2021), and exercise habits (Tuck et al., 2020).

As the other extreme of exercise behavior, exercise addiction tendency has also drawn research attention. Veale (1987) first put forward the concept, stating that exercise becomes a compulsive activity that occupies the core of daily life. Hausenblas and Downs (2002) further defined it as an intense craving for amateur sports, driving individuals to exercise excessively with physiological and psychological symptoms. A study by Çakır (2019) found a positive correlation between exercise frequency and exercise addiction tendency among health-major college students, with higher exercise frequency related to higher addiction risk. Although controversial, exercise addiction tendency is generally defined as a maladaptive psychological and behavioral pattern characterized by tolerance, withdrawal symptoms, and linked to psychological distress and injury risk (Terry et al., 2004). Its behavioral similarity to grit has triggered inquiries into their intrinsic relationship.

Previous studies mostly focused on a single dimension of grit or exercise addiction tendency. For example, Li et al. (2025) found that traditional and outdoor physical education help cultivate grit among college students, while Metin et al. (2023) reported that exercise addiction tendency is more common in students who exercise regularly. However, systematic studies combining both dimensions are rare. In the context of higher education, how to promote grit and prevent exercise addiction tendency through scientific exercise interventions remains an unsolved problem. To our knowledge, this study is the first specialized research on the differences and associations between grit and exercise addiction tendency among college students.

Accordingly, this study took freshmen and sophomores from China University of Geosciences (Wuhan) as participants, set 35 mandatory running sessions in one semester as the intervention reference standard, and adopted a prospective observational design. It empirically analyzed between-group differences in exercise addiction tendency and grit among students with different numbers of completed running sessions (< 35, = 35, > 35) and clarified their intrinsic correlation characteristics. By systematically exploring the nature of the relationship between exercise addiction tendency and grit, this study provides scientific empirical evidence for optimizing physical and mental health intervention strategies and improving academic achievement and quality of life among college students.

## 2 Methods

### 2.1 Participants

A total of 1,917 questionnaires were collected before observation, and 1,713 after observation. Considering potential invalid data such as incomplete responses, logical contradictions, or random answers, two investigators independently screened the data against strict criteria and then summarized the results. Finally, 1,611 valid questionnaires (930 males, 681 females, aged 18–22 years) from pre- and post-observation surveys were obtained for subsequent data analysis and conclusion derivation (Table 1).

**Table 1:** Basic characteristics of participants.

Total	Male	Female	Age (M ± SD)	Height/cm (M ± SD)	Weight/kg (M ± SD)	Grade
1,611	930	681	20.0 ± 2.0	171.5 ± 22.5	75.2 ± 31.4	Freshmen, Sophomores

Data were independently double-screened, yielding 1,611 valid questionnaires. Age, height, and weight are presented as mean ± standard deviation.

### 2.2 Observation procedure

This study targeted freshmen and sophomores from China University of Geosciences (Wuhan), with 35 mandatory running sessions in one semester (16 weeks) set as the observation compliance standard. This frequency was determined based on the fact that the consolidation of exercise habits requires repeated training over a certain period. Kaushal and Rhodes (2015) longitudinally confirmed that ≥ 4 sessions per week for 6 consecutive weeks (total 24–32 sessions) is the minimum threshold for exercise habit formation; 35 sessions slightly exceed this critical value to better ensure stable habit consolidation. Lally et al. (2010) proposed that habit formation requires an average of 66 days of repeated practice, and the 35-session setting within 16 weeks fits college students’ daily exercise rhythm and behavioral automation rules. In line with college physical education practice, 35 sessions fall within the median range of 30–40 sessions, the common assessment benchmark for extracurricular mandatory running in domestic universities, complying with practical standards for extracurricular physical exercise and effectively distinguishing three groups (under-target, target, over-target) to meet statistical grouping requirements.

Exercise requirements: 1.5 km per run for males and 1.2 km for females, with a pace of 3–9 min/km. Valid recording periods were 6:00–7:40 and 16:00–22:00 daily. Exercise data were recorded via the Budao Le-Pao APP (Le-Pao Sports Internet Co., Ltd., 2019), and overtime runs were not counted as valid sessions.

To ensure data authenticity, the research team developed a supporting mini-program and deployed face recognition systems on 6 designated running routes to conduct 24-h secondary monitoring for each running record. Abnormal records (e.g., vehicle-assisted running, cheating) were automatically judged invalid. Combined with the campus-wide internal network and school encrypted cloud server, the secure upload and storage of exercise data were guaranteed.

The questionnaire was developed via Wenjuanxing (Huang, February 19, 2025), verified by Zhou (February 22, 2025), and pre-tested among 87 college students on March 1, 2025. It was optimized based on feedback to form the formal questionnaire. The questionnaire was administered via QR codes announced by instructors during corresponding periods on March 27, 2025 (the first class before running started) and June 22, 2025 (the last class after running ended). Participants completed it independently, and the questionnaire included the Grit-O scale, EAI scale, and demographic questions. Running data were extracted from the Le-Pao backend on June 23, 2025.

### 2.3 Measurement tools

The Exercise Addiction Inventory (EAI) developed by Terry et al. (2004) was used as the assessment tool. The scale covers 6 core dimensions: importance, mood regulation, tolerance, withdrawal syndrome, impulsiveness, and relapse tendency. A 5-point Likert scoring method was adopted: strongly agree (5), agree (4), neutral (3), disagree (2), strongly disagree (1), with a total score ranging from 6 to 30 (6 items × 1 = 6, 6 items × 5 = 30; no scores below 6). The criteria for classifying exercise addiction based on EAI scores are as follows: 6–12 points = no exercise addiction characteristics; 13–23 points = presence of exercise addiction symptoms; ≥ 24 points = presence of exercise addiction, with higher scores indicating more severe addiction tendency. In this study sample, the pre-test internal consistency coefficient of EAI was *α* = 0.786, and post-test *α* = 0.763, both above the acceptable standard of 0.7, indicating good internal consistency and measurement reliability of the scale in this sample.

The Original Grit Scale (Grit-O) consists of 12 items using a 5-point Likert scale (1 = not like me at all, 5 = very much like me), with two dimensions: consistency of interest and perseverance of effort (Duckworth et al., 2007). Items include the ability to persist in adversity (e.g., “I have overcome a major challenge by persevering through setbacks”) and consistency of long-term interests (e.g., “I have difficulty focusing on projects that take several months to complete”). The internal consistency coefficients of Grit-O pre-test and post-test were 0.824 and 0.817, respectively, both above 0.70, indicating good internal consistency and reliability. In 2009, Duckworth and Quinn (2009) revised Grit-O by removing 4 items to form the 8-item Short Grit Scale (Grit-S), with confirmatory factor analysis showing good model fit. In 2018, Zhang (2018) revised the Chinese versions of both Grit-O and Grit-S and tested their validity among college students, confirming that Grit-O has better structural validity and is more suitable for measuring grit among Chinese college students, providing a reliable tool for assessing grit level in this population.

### 2.4 Ethical guidelines and privacy protection

This study strictly complied with academic ethical guidelines and was approved by the Ethics Committee of China University of Geosciences (Wuhan), covering research design, biometric data processing, privacy protection, and informed consent procedures. All research activities were conducted within the approved scope. The face recognition system was only used to verify the authenticity of running records, collecting only facial feature snippets during running without full facial information or association with personal identity. Relevant data were stored in the school’s dedicated encrypted cloud server using AES-256 encryption, isolated from the external network and protected from third-party disclosure. Access required dual authentication with full traceability. Facial feature snippets were automatically deleted within 24 h after abnormal judgment, and all data were completely deleted under the supervision of the ethics committee after the study with records retained. Before filling out the questionnaire, participants read the informed consent statement clarifying that names were only used for data matching, data were anonymized, participants could withdraw without responsibility at any time with data deleted promptly, etc. Voluntarily scanning the QR code to complete the questionnaire was deemed consent, fully protecting participants’ privacy and legitimate rights and interests.

### 2.5 Data analysis

Data analysis was performed on 1,611 participants divided into < 35 sessions (*n =* 380), = 35 sessions (*n =* 1,132), and > 35 sessions (*n =* 99) groups, with *α* = 0.05 set as the significance level. The Shapiro–Wilk test was used to verify normality of pre-test, post-test, and difference scores of EAI and Grit-O. The Levene test examined homogeneity of variance; standard ANOVA was used for homogeneous data, and Welch ANOVA for heterogeneous data. With the number of running sessions as the between-subject variable and measurement time as the within-subject variable, mixed-design ANOVA tested main effects of group, main effects of time, and time × group interaction effects. Tukey HSD was used for post-hoc tests of homogeneous data, and Games-Howell for heterogeneous data. Effect sizes were evaluated by *η*^2^ and Cohen’s d.

Within-group correlation, partial correlation after controlling for group, and multiple regression were applied to correct the correlation between EAI and Grit-O, eliminating inflation effects from between-group differences. The number of running sessions was treated as a continuous variable for correlation and polynomial regression in sensitivity analysis to test the dose–response relationship. Cronbach’s *α* coefficient was used to examine the internal consistency of the scales.

## 3 Results

Shapiro–Wilk normality tests were performed on the total pre-test and post-test scores of the EAI and Grit-O, as well as the pre–post difference scores. The results showed that only the pre-test, post-test, and difference scores of the EAI in the >35 sessions group were normally distributed (*p* > 0.05), whereas data for the other groups and all groups of the Grit-O were non-normally distributed (*p* < 0.001). Given the robustness of ANOVA to violations of normality in large samples, parametric tests remained appropriate (Table 2).

**Table 2:** Normality test results (Shapiro–Wilk).

Scale type	Group	Pre-test (W value/*p-*value)	Post-test (W value/*p-*value)	Difference (W value/*p-*value)	Normality judgment
Exercise Addiction Inventory (EAI)	<35 sessions	0.9507/<0.001	0.9362/<0.001	0.9830/0.072	Difference conforms
	=35 sessions	0.9490/<0.001	0.9693/<0.001	0.9867/0.001	None conforms
	>35 sessions	0.9831/0.833	0.9688/0.377	0.9553/0.143	All conforms
Grit Scale (Grit-O)	<35 sessions	0.9350/<0.001	0.9406/<0.001	0.4869/<0.001	None conforms
	=35 sessions	0.9320/<0.001	0.9376/<0.001	0.4273/<0.001	None conforms
	>35 sessions	0.9580/0.175	0.9573/0.165	0.7421/<0.001	Difference does not conform

Levene’s test for homogeneity of variance indicated that the pre-test and difference scores of the EAI showed homogeneous variance (p > 0.05), while post-test scores showed heterogeneous variance (*p* < 0.05). All pre-test, post-test, and difference scores of the Grit-O exhibited heterogeneous variance (p < 0.05). In subsequent analyses, standard ANOVA was used for data with homogeneous variance, and Welch ANOVA was applied for data with heterogeneous variance (Table 3). The descriptive statistics for the pre- and post-tests on the two scales for each subgroup are shown in the figure (Table 4).

**Table 3:** Variance homogeneity test results (levene).

Scale type	Pretest (*F*-value/*p*-value)	Post-test (F-value/*p*-value)	Difference (F-value/*p*-value)	Homogeneity of variance test
Exercise Addiction Inventory (EAI)	0.2604/0.7708	3.1533/0.0434	1.0261/0.3590	Homogeneous in pretest/difference, heterogeneous in posttest
Grit Scale (Grit-O)	3.6308/0.0271	4.0288/0.0183	3.1509/0.0435	Nonuniformity

A *p* > 0.05 indicates homogeneity of variance, and ANOVA is used; a *p* < 0.05 indicates heterogeneity of variance, and Welch ANOVA correction is applied.

**Table 4:** Descriptive statistical results of major variables (mean ± standard deviation).

Scale type	Divide into groups	Sample size (*n*)	Pretest (M ± SD, Min-Max)	Post-test (M ± SD, Min-Max)	Difference (M ± SD, Min-Max)
Addiction Inventory (EAI)	<35 times	380	14.89 ± 3.72 (8–30)	15.33 ± 3.92 (7–30)	0.44 ± 2.86 (−8–10)
	=35 times	1,132	15.39 ± 3.72 (7–30)	15.70 ± 3.33 (8–30)	0.31 ± 2.54 (−7–9)
	>35 times	99	18.95 ± 3.84 (11–28)	18.86 ± 2.62 (12–24)	−0.09 ± 2.31 (−6–7)
Grit-O Scale	<35 times	380	32.17 ± 3.92 (20–41)	32.40 ± 3.92 (20–42)	0.23 ± 3.15 (−8–10)
	=35 times	1,132	32.10 ± 4.37 (12–48)	32.18 ± 4.15 (15–49)	0.08 ± 3.02 (−9–8)
	>35 times	99	36.05 ± 5.51 (24–44)	37.00 ± 5.46 (24–45)	0.95 ± 3.28 (−5–12)

M ± SD denotes mean ± standard deviation; Min-Max indicates the minimum to maximum score on the scale; EAI total score ranges from 6 to 30 points, while Grit-O total score ranges from 12 to 60 points.

Tests of between-group main effects revealed significant group differences in pre-test scores for both scales (EAI: p < 0.05; Grit-O: p < 0.001). Appropriate post-hoc tests (Tukey HSD for homogeneous variance, Games-Howell for heterogeneous variance) showed that pre-test EAI and Grit-O scores in the >35 sessions group were significantly higher than those in the <35 sessions and = 35 sessions groups (*p* < 0.05), with no significant difference between the latter two groups (*p* > 0.05). This suggests that the high-frequency running group had significantly higher baseline exercise addiction tendency and grit (Tables 5, 6).

**Table 5:** Post-test results between pre-test groups of the exercise addiction scale (EAI) (Tukey HSD).

Comparison group	Mean difference	Standard error	*p-*value	95% confidence interval	Significant
<35times vs. = 35times	0.5051	0.3326	0.3382	[−0.3386, 1.3487]	Not significant
<35 times vs. > 35 times	4.0571	0.5892	<0.001	[2.4424, 5.6717]	significant
=35 times vs. > 35 times	3.552	0.5568	<0.001	[2.0510, 5.0530]	significant

Statistical results: *F*(2,607) = 17.9075, *p* < 0.001, *η*^2^ = 0.0557.

**Table 6:** Post-test results between pre-test groups of Gray-O (Games-Howell).

Comparison group	Mean difference	Standard error	df	*p-*value	95% confidence interval	Significant
<35 times vs. = 35 times	−0.07	0.3215	289.63	0.8458	[−0.9423, 0.8023]	Not significant
<35 times vs. > 35 times	−3.88	0.7265	52.17	0.0002	[−5.6012, −2.1588]	significant
=35 times vs. > 35 times	−3.81	0.6892	55.39	0.0001	[−5.4527, −2.1673]	significant

*p < 0.05, **p < 0.01, ***p < 0.001.

Paired-samples *t*-tests for within-group main effects showed no significant pre–post differences in EAI scores across all three groups (*p* > 0.05), with small effect sizes. For the Grit-O, marginally significant pre–post differences were found in the >35 sessions and ** < 35 sessions** groups (*p* ≈ 0.05), whereas no significant difference was observed in the ** = 35 sessions** group (*p* > 0.05). The effect size was larger in the >35 sessions group, indicating an improving trend in grit among the high-frequency running group (Tables 7, 8).

**Table 7:** Results of paired *t*-tests before and after the exercise addiction scale (EAI).

Groups	*t-*value	df	*p-*value	Cohen’s d	Result evaluation
<35 times	−1.104	143	0.2715	0.1164	Not significant
=35 times	−1.4453	428	0.1491	0.0871	Not significant
>35 times	0.1086	36	0.9141	0.0247	Not significant

Cohen’s d 0.2 = small effect, 0.5 = moderate effect, 0.8 = large effect.

**Table 8:** Results of paired *t*-tests before and after Gray-O scale.

Groups	*t -*value	df	*p-*value	Cohen’s d	Result evaluation
<35 times	−1.7203	143	0.0875	0.0585	Marginal significance
=35 times	−0.6911	428	0.4899	0.0197	Not significant
>35 times	−2.0003	36	0.0531	0.1725	Marginal significance

Significant edges indicate *p* = 0.05; Cohen’s d values of 0.2 = small effect, 0.5 = moderate effect, and 0.8 = large effect.

Between-group analyses based on pre–post differences revealed that the time × group interaction effect was not significant for the EAI (*p* > 0.05), indicating a consistent pattern of change in addiction tendency across groups with different running frequencies. The interaction effect was significant for the Grit-O (*p* < 0.05). Post-hoc tests showed a marginally significant difference in change scores between the >35 sessions and = 35 sessions groups (*p* ≈ 0.05), and the improvement magnitude in the >35 sessions group was significantly greater than in the other two groups. This indicates that running frequency significantly moderated the improvement in grit (Table 9).

**Table 9:** *Post-hoc* test results for difference groups on the Grit-O scale (Games-Howell).

Comparison group	Mean difference	Standard error	df	*p*-value	95% confidence interval	Significant
<35 times vs. = 35times	0.15	0.2893	291.45	0.8672	[−0.6487, 0.9487]	Not significant
<35 times vs. > 35 times	−0.72	0.6582	51.83	0.4329	[−2.3015, 0.8615]	Not significant
=35 times vs. > 35 times	−0.87	0.6291	54.76	0.0849	[−2.3921, 0.0521]	Marginal significance

Statistical results: Welch *F*(2,49.87) = 3.2845, *p* = 0.0452.

Within-group correlation, partial correlation, and multiple regression analyses confirmed that the correlation coefficients between the two scales within the three groups ranged from 0.087 to 0.189, and the partial correlation coefficients after controlling for group ranged from 0.076 to 0.083. Neither the main effect of EAI nor its interaction with group was significant (*p* > 0.05), indicating that the correlation was not inflated by between-group differences (Tables 10, 11).

**Table 10:** Correlation analysis results of EAI scale and Grit-O scale within each group.

Scale time points	Groups	Correlation coefficient r	*p*-value	95% confidence interval	Sample size (*n*)
Before measurement	<35 times	0.123	0.145	[−0.042, 0.282]	380
	=35 times	0.087	0.078	[−0.003, 0.175]	1,132
	>35 times	0.156	0.362	[−0.168, 0.445]	99
After test	<35 times	0.109	0.198	[−0.057, 0.269]	380
	=35 times	0.092	0.061	[0.002, 0.180]	1,132
	>35 times	0.189	0.271	[−0.132, 0.473]	99

r > 0.3 indicates weak correlation, while r > 0.5 indicates moderate correlation; 95% CI denotes the 95% confidence interval; *p* < 0.05 signifies statistically significant correlation.

**Table 11:** Partial correlation analysis results of EAI scale and Grit-O scale after control group division.

Scale time points	Partial correlation coefficient r_p	*p-*value	95% confidence interval	Controlled variable
Before measurement	0.076	0.089	[−0.008, 0.159]	Group
After test	0.083	0.063	[−0.001, 0.165]	Group

*p* < 0.05 indicates significant partial correlation.

In the sensitivity analysis, running frequency treated as a continuous variable was significantly and weakly positively correlated with scores on both scales (*p* < 0.001). Polynomial regression supported a linear dose–response relationship, suggesting that increases in running sessions were associated with gradual linear rises in both indicators (Table 12).

**Table 12:** Correlation analysis between running frequency and scale scores.

Correlativity	Correlation coefficient r	*p-*value	95% confidence interval
Number of runs vs. pre-test EAI	0.228	<0.001	[0.156, 0.296]
Number of runs vs. pretest grit	0.243	<0.001	[0.172, 0.311]
Number of runs vs. post-test EAI	0.196	<0.001	[0.124, 0.265]
Number of runs vs. post-test grit	0.215	<0.001	[0.144, 0.283]

*p* < 0.001 indicates extremely significant correlation; r denotes the Pearson correlation coefficient.

Cronbach’s *α* coefficients were 0.786 (pre-test) and 0.763 (post-test) for the EAI, and 0.824 (pre-test) and 0.817 (post-test) for the Grit-O, all exceeding the threshold of 0.7. This indicates good internal consistency and reliable measurement for both scales (Table 13).

**Table 13:** Results of internal consistency reliability analysis for the scale (Cronbach’s *α*).

Scale type	Time point	Cronbach’s *α*	95% confidence interval	Number of entries	Calibrated *α*
Addiction Inventory (EAI)	Before measurement	0.786	[0.754, 0.815]	6	0.762–0.798
	After test	0.763	[0.729, 0.794]	6	0.738–0.775
Grit Scale (Grit-O)	Before measurement	0.824	[0.801, 0.845]	12	0.811–0.836
	After test	0.817	[0.793, 0.838]	12	0.803–0.829

An alpha coefficient > 0.7 indicates good internal consistency of the scale.

## 4 Discussion

The results of this study indicate no significant pre-post differences in EAI scores across the three groups, while only the > 35 and < 35 sessions groups show marginally significant improvements in Grit-O scores. The > 35 sessions group has significantly higher baseline addiction tendency and grit than the other two groups, and the number of running sessions significantly moderates grit improvement, whereas the time × group interaction effect for EAI is not significant. The weak correlation between exercise addiction tendency and grit is attributed to between-group differences with no substantial intrinsic association, yet the number of running sessions shows a weak linear positive correlation with both indicators.

Based on the above findings, the discussion focuses on three aspects: first, the potential causes of psychological trait differences among running groups; second, the moderating effect of running frequency and differences in motivational mechanisms; third, the essential nature and research value of the relationship between exercise addiction tendency and grit.

One major contribution of this study is that, using 35 running sessions as the compliance cutoff, it reveals that students exceeding this threshold have significantly higher exercise addiction tendency and grit than those failing to meet or exactly meeting the standard—a topic not previously explored. Previous studies consistently support a positive association between grit and physical activity participation. Hromyak et al. (2025) found that individuals with higher grit tend to engage in and adhere to physical activities. Lee et al. (2022) noted that athletes with higher grit are more likely to invest more time in sports, and grit enhances their motivation when facing obstacles, promoting exercise adherence. The significantly higher grit scores in the > 35 sessions group in this study are highly consistent with the above conclusions, reaffirming grit as a key personality trait for long-term goal achievement.

Grit is defined as passion and perseverance for long-term goals (Eskreis-Winkler et al., 2014). Pre-test results show baseline differences in grit across groups, suggesting that students with higher pre-test grit inherently possess stronger perseverance and resilience, facilitating high motivation and implementation in long-term exercise programs to complete or exceed the 35-session target. The formation of exercise addiction tendency is closely related to exercise frequency and intensity (Üzgü et al., 2023); the pleasure and achievement from frequent exercise may trigger stronger dependence, leading to higher addiction scores. In contrast, students with lower grit tend to abandon exercise due to academic pressure, time conflicts, etc. (Huéscar Hernández et al., 2020; Priskila et al., 2023), failing to meet exercise targets without deep engagement, resulting in lower addiction scores.

Another major contribution is confirming that running frequency significantly moderates grit improvement but does not affect the change pattern of addiction tendency. According to Self-Determination Theory (Ryan and Deci, 2000), human behavioral motivation lies on a continuum from external to internal. External regulation and introjected regulation are controlling and fail to sustain behavior. The behavior of the = 35 sessions group aligns more with external regulation, motivated mainly by course requirements and credit acquisition; students tend to stop exercising after meeting the target without internalizing exercise as a personal need, leading to lower grit and lower addiction risk.

In contrast, the > 35 sessions group exhibits intrinsic drive beyond task requirements. Standage et al. (2003) showed that athletes driven by intrinsic motivation experience higher enjoyment and longer participation, and such motivation internalization is the source of long-term passion in grit. For this group, long-term exercise adherence tempers willpower, and successful experiences overcoming obstacles enhance self-efficacy, resulting in significantly higher grit scores. However, exercise occupies a central role in their self-concept, and inability to exercise may trigger identity crisis and negative emotions—manifestations of withdrawal and conflict in exercise addiction, explaining their higher addiction scores.

Notably, addiction tendency is not moderated by session frequency, indicating its formation depends not merely on exercise frequency but on motivational nature, cognitive appraisal, and other complex factors (Sicilia et al., 2021). The high addiction scores in the > 35 sessions group may be a byproduct of excessive focus on intrinsic motivation rather than a direct result of frequency. The other two groups do not develop irrational exercise dependence, so their addiction tendency shows no significant changes. This reveals that the moderating effect of running frequency is trait-specific, only affecting grit requiring sustained volitional investment, while addiction tendency is constrained by more complex psychological mechanisms.

Within-group correlation, partial correlation, and multiple regression confirm that the weak correlation between exercise addiction tendency and grit stems from between-group differences with no substantial intrinsic association, clarifying a previous cognitive misunderstanding. Essentially, although the high-frequency running group shows both high addiction tendency and high grit, their behavioral driving logics are fundamentally different: grit centers on rational persistence and investment in constructive long-term goals (Zhi and Yang, 2024) toward self-growth, whereas addiction tendency reflects irrational dependence on exercise that may neglect other life domains (Lubecka et al., 2021). Their similar external behaviors easily lead to misjudgment of correlation. This study, through corrected analyses controlling for between-group differences, verifies this as a spurious correlation resulting from the superposition of baseline traits and behavioral frequency across groups.

## 5 Conclusion

Although exercise addiction tendency and grit in college students’ mandatory running show no substantial intrinsic correlation, both are significantly affected by the number of running sessions and follow differential developmental rules. The high-frequency running group (> 35 sessions) has a higher baseline grit level and exhibits an upward trend in grit during observation, while their exercise addiction tendency is also significantly higher than that of medium- and low-frequency groups. This emphasizes that educational practice should adhere to the principles of moderate participation and motivational guidance, encouraging students to moderately increase running frequency based on voluntary will to cultivate grit. Meanwhile, exercise cognitive education should be strengthened to prevent grit cultivation from being transformed into a trigger for exercise addiction, achieving constructive shaping of psychological qualities through physical exercise.

## 6 Limitations

This study has the following limitations: first, it failed to consider running intensity and individual differences among college students, such as training motivation (intrinsic/extrinsic), psychological characteristics (personality, self-efficacy, anxiety level), training intensity (running pace, single running duration), and other confounding variables, which may lead to certain biases in interpreting between-group differences. Second, it focused only on mandatory running as a single exercise form without distinguishing different sports, so the generalizability of conclusions requires further verification. Third, there was an imbalance in sample size across groups after grouping by 35 running sessions, with a relatively small sample in the > 35 sessions group, which may affect statistical power and stability of result extrapolation. Future studies can be optimized by expanding the total sample and balancing sampling size across groups.

## 7 Prospects

Future research can be carried out in the following aspects: first, strictly control confounding variables such as baseline physical activity level, exercise motivation, training intensity, and psychological characteristics through stratified sampling and matching design to further improve the reliability and accuracy of conclusions. Second, expand the sample scope to include college students from different regions and types of universities to enhance the generalizability of findings. Third, distinguish different sports to explore their effects on students’ grit and exercise addiction, providing theoretical basis and practical strategies for promoting scientific public fitness, student sports practice, and avoiding exercise dependence.

## Data Availability

The original contributions presented in the study are included in the article/supplementary material, further inquiries can be directed to the corresponding author.
